# Fungal outbreak in the Catacombs of SS. Marcellino and Pietro Rome (Italy): From diagnosis to an emergency treatment

**DOI:** 10.3389/fmicb.2022.982933

**Published:** 2022-11-10

**Authors:** Filomena De Leo, Irene Dominguez-Moñino, Valme Jurado, Laura Bruno, Cesareo Saiz-Jimenez, Clara Urzì

**Affiliations:** ^1^Department of ChiBioFarAm, University of Messina, Messina, Italy; ^2^Institute for Natural Resources and Agrobiology, Spanish National Research Council (IRNAS-CSIC), Seville, Spain; ^3^Department of Biology, University of Rome “Tor Vergata,” Rome, Italy

**Keywords:** Roman Catacombs, biodeterioration, Basidiomycetes, *Coniophora* species, fungal outbreak

## Abstract

The present study reports a sudden fungal outbreak that occurred in the corridor near the entrance of the Catacombs of SS. Marcellino and Pietro in Rome (Italy) observed after 1 year of a restoration treatment that interested the walls of the entrance of the Catacombs and some artifacts placed *in situ*. The colonization was observed on the vault at the entrance and in correspondence with the restored marble pieces displayed on the left side of the corridor. No growth was observed on the right side where similarly treated marble slabs were placed. Samples taken in correspondence with fungal biofilm were analyzed through the combined use of microscopical, cultural, and molecular tools and showed that the vault and the left side of the corridor entrance were colonized by a complex fungal biofilm consisting mainly of *Coniophora* sp. and other genera, such as *Hypomyces*, *Purpureocillium*, *Acremonium*, *Penicillium*, and *Alternaria*, many of which are well known as responsible of biodeterioration of stone surfaces. Regarding the brown-rot basidiomycete *Coniophora*, it was able to form very large colonies on the substrata with a diameter of up to 57 cm. Although the direct observation under a light microscope evidenced the presence of abundant brown fungal conidia, several attempts to cultivate the microorganism failed, therefore only through DNA sequencing analyses, it was possible to identify and characterize this fungus. There is very little literature on the genus *Coniophora* which is reported as one of the causes of wet-rot decay of wood in buildings. A connection with calcium-containing materials such as bricks and mortars was demonstrated, but no data were available about the possible role of this species in the biodeterioration of stones. This study features the first finding of a strain related to the basidiomycetous genus of *Coniophora* in the order Boletales in association with evident phenomena of biodeterioration.

## Introduction

Fungi are well known for their ability to colonize a plethora of substrata, both organic and inorganic due to their ubiquitous characteristics and their great adaptability to nutrient concentrations, either low or rich.

Hypogea, due to the physico-chemical characteristics of quite stable temperature, high relative humidity, and continuous availability of organic sources (derived from soil, dead animals, microorganisms, visitors, etc.) represent a favorable environment for microbial growth. However, as reported by Urzì et al. (2018), studies on fungi in hypogean environments are relatively scarce, especially in the cultural heritage context.

Vanderwolf et al. (2013) listed 1,029 fungal species in 518 genera that mainly belonged to the phylum Ascomycota found in caves and mines worldwide, underlining that the origin and ecological role of fungi in hypogean environments remain unknown although they are related to the ubiquitous, oligotrophic, and psychrotrophic characteristics of the species found.

The presence of fungi in hypogean environments has been often associated with evident phenomena of biodeterioration among which are listed white and gray alterations, black stains, and profuse mycelial growth (Dupont et al., 2007; Jurado et al., 2009, 2010; Bastian et al., 2010; Urzì et al., 2010; De Leo et al., 2012; Martin-Sanchez et al., 2012; Ma et al., 2015; Sugiyama et al., 2017; Zucconi et al., 2022). Sudden changes and/or fluctuation of microenvironmental parameters may cause spore germination with a consequent rapid, intense, and diffuse fungal colonization on plaster and frescoes (Caneva et al., 2005). These events of heavy fungal colonization were described several times in Roman hypogea and were attributed to the opening and closing of the doors to allow the entrance of visitors and/or restoration activities that boosted the dissemination of spores (Florian, 1996; Albertano et al., 2005). Similar events described as “fungal outbreak” were observed in the Lascaux Cave in France and Castañar de Ibor Cave in Spain (Dupont et al., 2007; Bastian et al., 2010; Jurado et al., 2010; Martin-Sanchez et al., 2014).

This study aimed to investigate the sudden and heavy fungal outbreak that occurred in the vault and corridor near the entrance of the Catacombs of SS. Marcellino and Pietro in Rome (Italy), observed 1 year after a restoration treatment that interested the walls of the entrance of the Catacombs and some marble artifacts placed *in situ*. This fungal growth could have compromised both the fruition by visitors due to the possible allergenic effect of airborne spores, and the state of conservation of the monument, therefore some emergency treatments were also suggested.

## Materials and methods

### Case study

#### Catacombs of SS. Marcellino and Pietro

**FIGURE 1:**
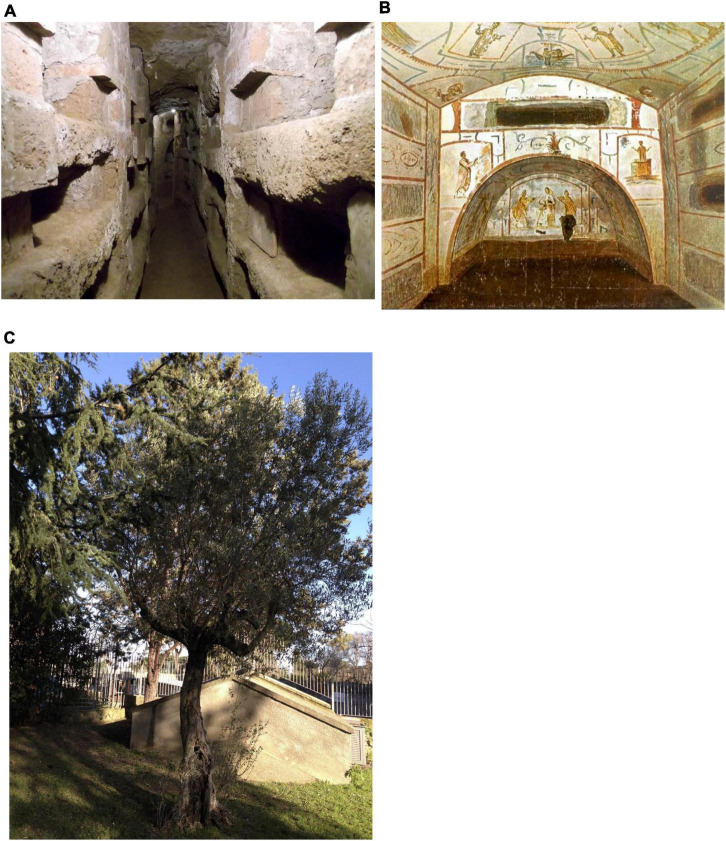
Overview of the SS. Marcellino and Pietro Catacombs in Rome. **(A)** Tomb excavated in the tupha; **(B)** cubicula (small room) with colorful frescoes; and **(C)** olive tree above the ground of the Catacombs.

The Catacombs of Saints Marcellino and Pietro were excavated between the third and fifth centuries A.C. and are also called the Catacombs of St. Elena or Catacombs of St. Tiburzio. They are one of the largest Catacombs situated on the third mile of the ancient *Via* Labicana, today *Via* Casilina in Rome, Italy, near the church of “SS. Marcellino and Pietro” ad Duas Lauros (at two laurels) (Figures 1A–D).

The Catacombs evolve in several corridors (Figure 1A), some of them still contain sealed niches with a skeleton inside, and in the past phototrophic biofilms as biodeteriogenic patinas have been described there (Bruno et al., 2019). Further, they contain several cubicula with high-quality frescoes (Figure 1B).

**FIGURE 2:**
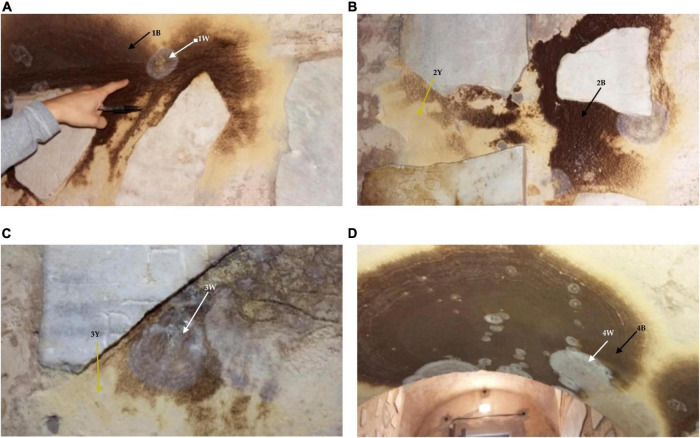
Macroscopic view of the fungal outbreak occurring on the wall and vault at the entrance of the Catacombs SS. Marcellino and Pietro. **(A)** Extensive brown and white/grayish fungal growth around a marble piece in correspondence of sampling point 1; **(B)** brown and white/grayish colonies around another marble piece in correspondence of sample 2; **(C)** brown and white/grayish fungal colonization on the sampling point 3; and **(D)** extended dark brown fungal colonization with secondary white/grayish colonies observed on the entrance vault in correspondence of sampling point 4. Arrows indicate the sampling points.

For this reason, they were chosen to be part of the Jubilee path of Mercy 2015–2016. Before the opening and thus to allow safer access to visitors, a new entrance was opened, and the Catacombs underwent a restoration intervention (2014–2015) for the implementation of the safety conditions of the Catacombs. At the end of Summer of 2015 on the walls and vault near the entrance, a sudden growth of black/brown colonies with a yellow halo was observed as well as the presence of smaller white/grayish colonies (Figures 2A–C).

### Sampling areas

The fungal biofilm observed on the Catacombs surfaces appeared as very large colonies of dark brown color with a diameter varying up to 50 cm and more than 120 cm on the vault, depending on the growth stage (Figures 2A,D). Sometimes smaller grayish-white colonies of considerably smaller size were observed on top of the dark large colonies. Fungal growth was also observed at the edge of marble slabs displayed on the left side of the entrance corridor (Figures 2A–C).

Relative humidity (RH) and temperature (°C) were recorded at the different parts of the Catacombs entrance and corridor, through a portable thermohygrometer Hanna Instruments HI18564, while irradiance was measured using a radiometer (model LI-185B; LI-COR inc., USA) equipped with a quantum sensor (LI-190SB) to give a measure of the PAR (Photosynthetic active radiation) in μmol photons m^–2^ s^–1^.

A multi-step approach consisting of airborne spores sampling, microscopy, and cultural and molecular analyses was carried out.

To assess the presence of airborne spores, adhesive tapes, commonly used for aerobiological analyses (Brighetti et al., 2014), were placed in small open Petri dishes near the contaminated areas for 24 h and then removed and observed at the light microscope Zeiss Axioscope. Experiments were performed in triplicate.

A total of 13 samples were taken from four different sites of the Catacombs indicated with numbers from 1 to 4, in correspondence of evident fungal colonization showing different pigmentation as brown (B), white (W), and yellow (Y) and morphology by using (a) the non-destructive sampling methods of adhesive tape strips (FungiTape© Did, Milan, Italy) (Urzì and De Leo, 2001) for carrying out microscopy and cultural analysis; and (b) a sterile scalpel for scraping little amounts of mycelium from lithic substrata for molecular analysis, as summarized in Table 1. In particular, four samples were taken from sites 1 and 2, three samples from sites 3, and two samples from site 4. Adhesive tape samples were preliminary cut into little squares of about 0.5 cm^2^ and used for microscopy and cultural analyses, while biofilm samples were analyzed through molecular methods.

**TABLE 1:** Samples were taken from the fungal biofilms on the wall and vault of the Catacombs, the size of colonies, and modality of sampling.

Samples	Morphology of fungal colonies on substrata	Diameter (cm)	Adhesive tape	Scalpel
1B	Brown with abundant aerial mycelium	57	+	+
1W	White/grayish, flat	9	+	+
2Y	Yellow biofilm, no aerial mycelium	47	+	+
2B	Brown with abundant aerial mycelium	7	+	+
3W	White/grayish flat	n.d.	+	−
3Y	Yellow biofilm no aerial mycelium	n.d.	+	+
4W	White/grayish flat	34	+	−
4B	Brown colony with abundant aerial mycelium	>120	+	−

### Microscopy

Samples taken with adhesive tape were directly observed under Light Microscopy equipped with phase contrast (LM, Leica DMLB Wetzlar, Germany). To this purpose, little squares of adhesive tape were placed face down on a glass slide with a drop of sterile water or Amman’s lactophenol solution and covered with a cover slide to keep the tape as flat as possible as described by Urzì and coworkers (Urzì and Albertano, 2001; Urzì and De Leo, 2001).

### Cultural analyses

Cultural analyses were carried out by placing a little square of adhesive tape as above specified onto the center of agar plates of Dichlorane Rose Bengal Chloramphenicol medium (DRBC, Oxoid, Basingstoke, UK) in duplicate. Malt Extract Diclorane Agar (MYBDA), LGBA Lignin Guaiacol Benomyl agar medium with Indulin AT (1 g/L), LGBA with Humic acid (1 g/L), and LGBA with Indulin AT (1 g/L), and Humic acid (1 g/L) (Thorn et al., 1996; Thompson, 1998; Thompson et al., 2012) were used when the first attempt to grow the oval-conidia producers in DRBC failed. Incubation was carried out at 26°C for up to 1 month.

Fungal isolates identification was carried out based on the macroscopic feature of colonies grown on different cultural media and the micro-morphology of reproductive structures, according to Ellis (1971, 1976), Barnett and Hunter (1972), Fassatiovà (1986), and de Hoog et al. (2019).

### Molecular analyses of samples

Molecular techniques were used for the evidence and identification of fungi from biofilm samples (1W, 1B, 2B, 2Y, and 3Y). No molecular analyses were carried out for sample 4 due to the similarity of the alteration observed and then confirmed under the microscope by the homogeneous presence of conidia comparable to those of samples 1 and 2. DNA was extracted using FastDNA SPIN Kit for Soil (MP Biomedicals, Illkirch, France). The fungal internal transcribed spacer regions were amplified by polymerase chain reaction (PCR) using the primers ITS1 (5′-TCC GTA GGT GAA CCT GCG G-3′) and ITS4 (5′-TCC TCC GCT TAT TGA TAT GC-3′) (White et al., 1990). PCR amplifications were performed in a Bio-Rad iCycler thermal cycler (Bio-Rad, Hercules, CA, USA) using the following thermal conditions: one cycle of denaturation (94°C for 2 min), followed by 35 cycles of denaturation (94°C for 1 min), annealing (50°C for 1 min), elongation (72°C for 10 min), and a terminal elongation step (72°C for 5 min) (Jurado et al., 2010). Amplification products were evaluated by electrophoresis on 1% (w/v) agarose gels, stained with SYBR Green I (Molecular Probes, Eugene, OR, USA), and visualized under UV light (Martin-Sanchez et al., 2014). The amplified PCR products were purified using the JetQuick PCR Purification Spin Kit (Genomed, Löhne, Germany). Purified products were cloned with a TOPO-TA Cloning Kit for Sequencing (Invitrogen, Carlsbad, CA, USA) and then were transformed into One Shot DH5α-T1 competent cells *Escherichia coli* (Invitrogen, Carlsbad, CA, USA), according to the manufacturer’s instructions. Selected clones for each sample were sequenced by Macrogen Inc. (Amsterdam, Netherlands). Fungal identification was performed using the BLASTn algorithm (Altschul et al., 1990) to the non-redundant databases of sequences deposited at the National Center for Biotechnology Information (NCBI). The sequences were deposited into the GenBank database under accession numbers which are as follows: ON5101061 and ON510107-ON510111. For phylogenetic analyses, sequences were retrieved from the GenBank database that is accessible from NCBI platforms.^1^ Alignments were created using the multiple sequence alignment program MUSCLE (Edgar, 2004). Molecular Evolution Genetic Analyses version 11 was employed for phylogenetic tree construction (Tamura et al., 2021). The evolutionary history using the ITS region sequences was inferred by using the máximum-likelihood method, Kimura’s two-parameter model with a discrete Gamma distribution, and invariant sites (Kimura, 1980). The robustness of the tree was evaluated by bootstrap resampling (1,000 replicates).

## Results and discussion

**FIGURE 3:**
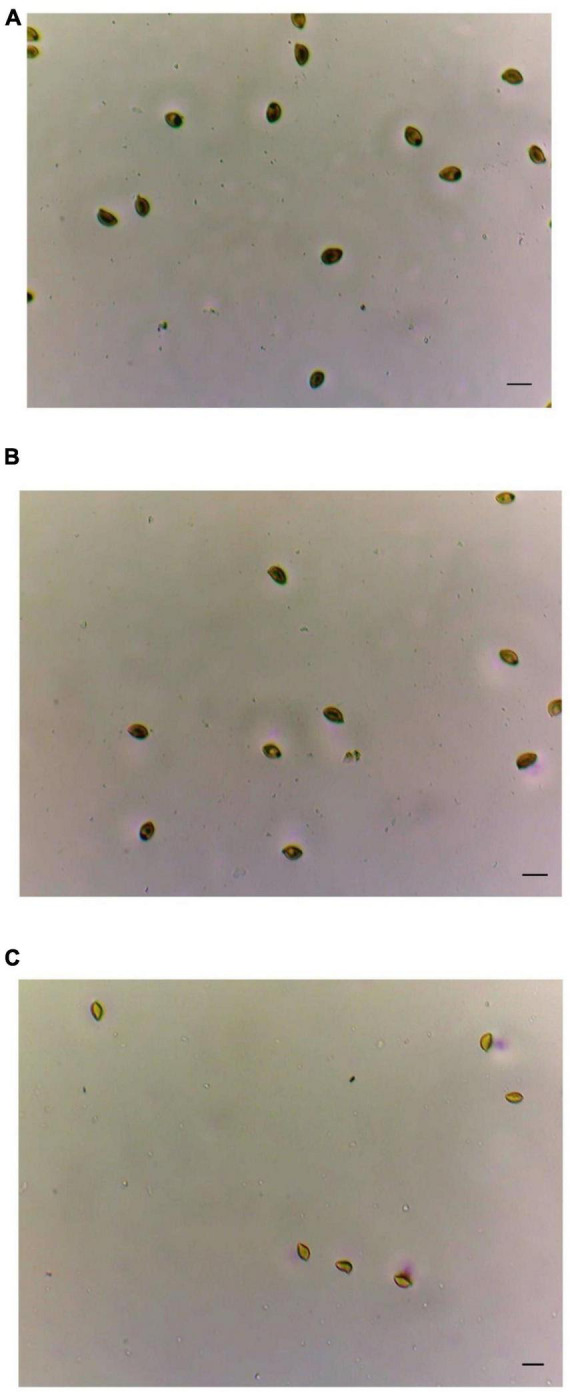
Quali/quantitative determination of airborne conidia taken after 24 h of exposure to adhesive tapes for aerobiological analysis. **(A)** At the entrance; **(B)** at the beginning of the corridor; and **(C)** toward the end of the corridor. In all the samples, the number of airborne spores/24 h was quite homogeneous in the studied environment in each microscopic area examined. Bar is 10 mm.

**FIGURE 4:**
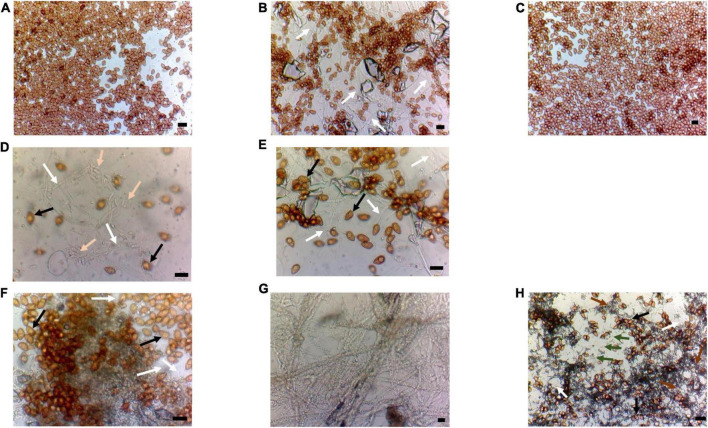
Adhesive tape samples were taken in correspondence with brown colonies and yellow and white colonies as shown in Figure 2. **(A–C)** Brown colonies. **(A)** Sample 1B; **(B)** sample 2B; and **(C)** sample 4B. Morphological observations suggest the presence of homogeneous oval Basidiomycetes conidia; in panel **(b)** are also visible hyaline hyphae (indicated by white arrows). **(D,E)** Yellow biofilm. **(D)** Sample 2Y; and **(E)** sample 3Y; it is observed the contemporary presence of black conidia and hyaline one (*Fusarium*-like) and hyaline hyphae (indicated by black, pink, and white arrows, respectively); **(E)** only black conidia and hyaline hyphae (indicated by black and white arrows, respectively). **(F–H)** White/grayish colonies. **(F)** The abundant presence of black ovoidal conidia and hyaline hyphae in the background (indicated by black and white arrows, respectively); **(G)** only hyaline hyphae; and **(H)** are visible two kinds of conidia (black ovoidal and roundish this latter attributable to *Aspergillus niger* indicated by black and green arrows, respectively) as well as hyaline and melanized hyphae (indicated by white and brown arrows, respectively). Bar is 10 mm.

**FIGURE 5:**
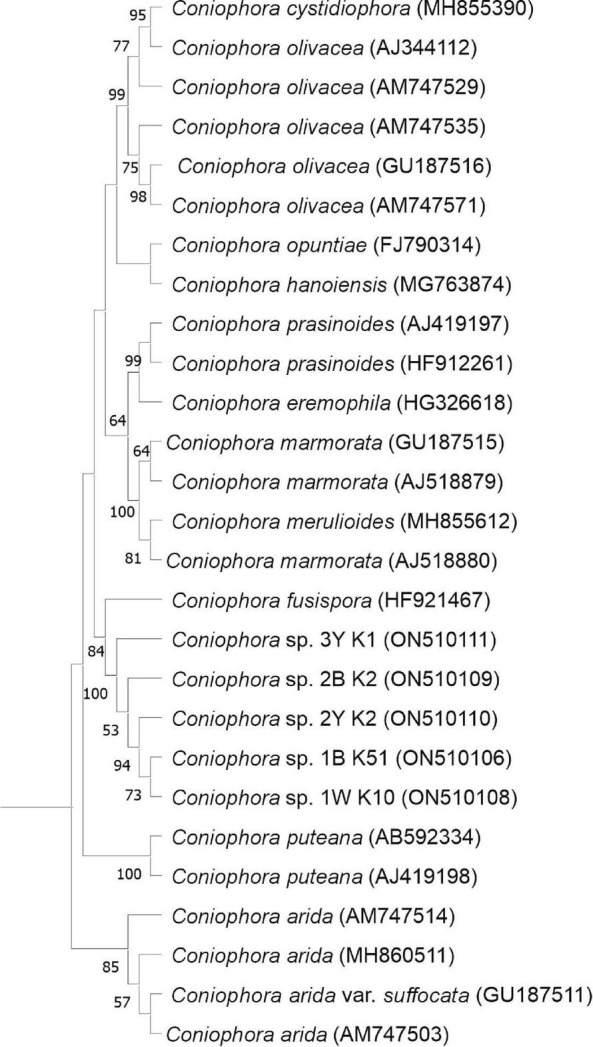
Maximum-likelihood phylogenetic tree based on ITS region sequences showing the relationship of *Coniophora* species. Bootstrap values (>50%) are expressed as percentages of 1,000 replicates. The sequence of *Actinomyces oris* (NR104896) was used as an outgroup.

The results obtained are summarized in Tables 2, 3 and Figures 3–5.

**TABLE 2:** Environmental parameters measured at the four sampling sites.

SITE	PAR μ mol m^–2^ s^–1^	RH%	T°
1	0.1–0.4	86	21.9
2	0.5	86.1	21.9
3	1.2	86.2	21.8
4	0.1–0.3	86.9	21.8

**TABLE 3:** Nearest neighbor rDNA sequences of *Coniophora marmorata* and references from NCBI databases and bibliographic references.

Sample	Representative clone	No. of clones	Closest relative sequences and % of similarity	References
1B	1B_K51	9	*Coniophora marmorata* 83.07% AJ518879	Schmidt et al., 2002; Schmidt and Moreth, 2002
	1W_K10	4	*Coniophora marmorata* 84.63% AJ518879	Schmidt et al., 2002; Schmidt and Moreth, 2002
2B	2B_K2	9	*Coniophora marmorata* 83.36% AJ518879	Schmidt et al., 2002; Schmidt and Moreth, 2002
2Y	2Y_K2	8	*Coniophora marmorata* 84.05% AJ518879	Schmidt et al., 2002; Schmidt and Moreth, 2002
3Y	3Y_K1	8	*Coniophora marmorata* 84.13% AJ518879	Schmidt et al., 2002; Schmidt and Moreth, 2002

The microclimatic parameters evidenced in all the sampling sites are quite uniform values regarding RH%, T°, and light intensity as shown in Table 2.

The aerobiological analyses reported a constant presence in the air of black conidia whose findings were also confirmed by the consequent contamination of the surfaces seen under the microscope observation (Figure 3).

In particular, the slides observed for the aerobiological qualitative determination of the air quality as well as the adhesive samples taken from the brown colony (1B, 2B, 3B, and 4B) showed the almost exclusive presence, widespread and numerous, of fungal conidia with a morphology that is compatible with conidia of fungal species belonging to the phylum Basidiomycota. Microscopic observation of samples taken from the different colonies such as those described as yellow (Y) or white/grayish (W) showed the coexistence of hyaline hyphae and ovoid dark-colored spores confirming the spread of fungal spores of Basidiomycetes (Figure 4) as well as the presence of secondary colonization due to the ubiquitous Ascomycetous genera of *Aspergillus*, *Penicillium, Alternaria, Fusarium*, and *Paecilomyces*, as confirmed by the findings of cultural analysis (Supplementary Figure 1 and Supplementary Table 1). No fungal strains associated with the black conidia observed under direct microscopy have grown in the cultural media.

Only through molecular analyses of biofilm samples, it was possible to demonstrate the dominant presence of basidiomycetous species related to the brown-rot fungal genus *Coniophora.* The sequence similarity search showed that the closest species belonged to the *Coniophora* genus with a low percentage of identity (in all cases less than 86.92%) and, in particular, the nearest sequences of *Coniophora marmorata* (AJ518879), the only *Coniophora* species known to be able to grow on rocks, mortars, and bricks, was ranging from 83.07 to 84.63% of sequence identity (Table 3 and Figure 5). These low percentages are evidence that the fungal strains causing the outbreak in the Catacombs of SS. Marcellino and Pietro most likely represent a new species. However, efforts to culture the fungus in the laboratory failed, despite the wide range of culture media used. The clone 1W K2 (ON 510107) representative of five clones, resulted near to the specie of *Hypomyces chlorinigenus* (93.23% KT946843, Otto et al., 2016).

## Discussion

Our analysis demonstrated that the vault and the left side of the corridor at the entrance of the Catacombs of SS. Marcellino and Pietro were heavily affected by fungal colonization mainly due to a strain related to the basidiomycetous genus of *Coniophora* in the order Boletales. In further support of this attribution, the presence of the ascomycetous strains belonging to the genus *Hypomyces*, which are well-known parasites of *Boletales* species, was evidenced (Rogerson and Samuels, 1989). Members of the genus *Coniophora*, among which *C. puteana* and the less common species *C. marmorata*, *C. arida*, and *C. olivacea*, the so-called “cellar fungi” are known responsible for wood brown-rot in indoor and outdoor buildings structures (Schmidt, 2007; Gabriel and Švec, 2017). Moreover, *C. marmorata* has been associated with calcareous materials such as bricks and mortars (Singh, 1994) but to our knowledge, its biodeteriorative activity on stone monuments has never been demonstrated.

The *Coniophora* species are very difficult to distinguish from each other, and traditional cultural methods fail to grow this fungus. Thus, molecular techniques were applied to obtain a reliable method for their differentiation. However, the results obtained were not conclusive; due to the low percentage of rDNA sequence similarity, we may even suppose that the strain associated with the outbreak could belong to a not yet identified species. Further studies are in progress both to attempt the cultivation of the fungus that would allow it to deepen its physiology and taxonomy, and to investigate the possible sources of contamination. Although, no decaying wood was observed in the studied site, the presence of protruding roots of an olive tree situated above the ground in the proximity of the fungal colonization was observed (Figure 1C). Further inspection of the tree did not show any apparent illness at the time of the study, but this does not exclude the tree or its roots as a possible source of contamination.

Regarding their risk potential to health, the existing literature is not vast and only a few studies (Helbling et al., 1999) have shown cases of allergic reactions due to Basidiomycetes. So, although these are not notoriously allergenic microorganisms, it is not possible to completely exclude the risk for operators and visitors. Given the extent of macrocolonies, their fast growth rate, and the spread of spores in high quantities, we suggested an emergency intervention aimed at reducing the colonization process and at the same time decreasing the risk of allergic reactions and/or asthma attacks both toward operators and visitors, especially for those subjects with reduced immune defenses, such as the elderly, children, and people with respiratory diseases.

The treatments foreseen are (a) intervention on the environment by continuous monitoring of the level of circulating spores and appearance of newly formed colonies; and (b) biocide treatments direct to the fungal colonies. Due to the fact that several causes could have created this fungal outbreak, continuous monitoring of the Catacomb’s surfaces is still undergoing.

## Data availability statement

The datasets presented in this study can be found in online repositories. The names of the repository/repositories and accession number(s) can be found in the article/Supplementary material.

## Author contributions

FD, LB, CU, and CS-J: conceptualization. LB, FD, ID-M, and VJ: methodology. FD, CU, and VJ: resources. FD, LB, CU, CS-J, and VJ: data curation, writing—original draft preparation and review, and editing. All authors have read and agreed to the published version of the manuscript.

## Footnotes

1 http://www.ncbi.nlm.nih.gov, accessed on 9 May 2022 and 26 August 2022.
