# Supplementary material for: Fungal outbreak in the Catacombs of SS. Marcellino and Pietro Rome (Italy): From diagnosis to an emergency treatment
Source: Front Microbiol. 2022 Nov 10;13:982933. doi: 10.3389/fmicb.2022.982933 (PMC9684309; doi:10.3389/fmicb.2022.982933)
Supplement: Supplementary file 1 [file Image_1.pdf]

Figure 1S. Results of growth of fungal colonies from samples taken with adhesive tape and inoculated on the isolation DRBC agar medium. The tentative identification of colonies is reported in the Table 1S. Petri dishes with no growth in both replicates are not reported.

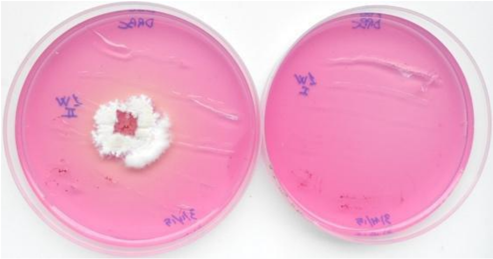
1W

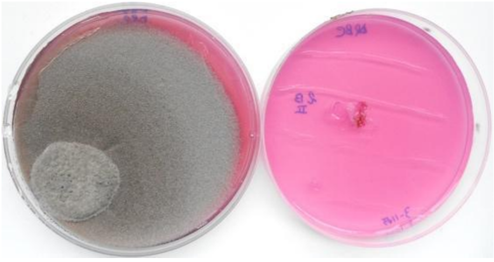
2B

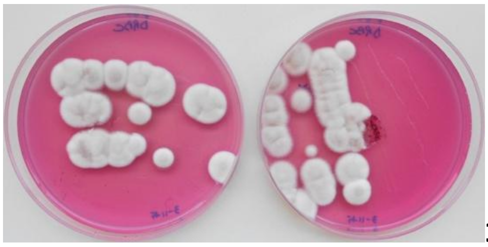
3W

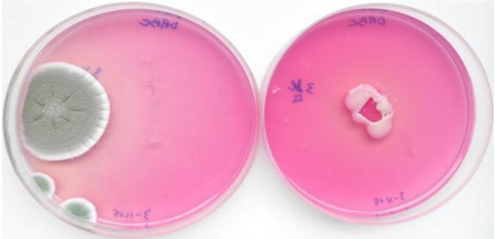
3Y

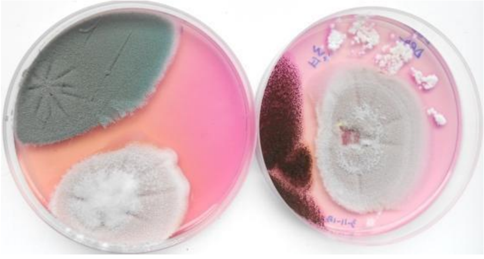
4W

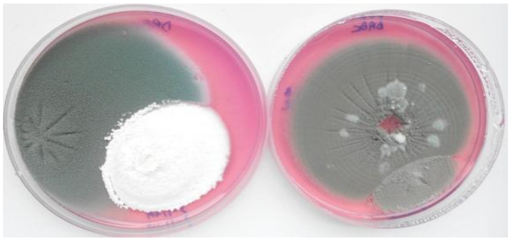
4B
