# Supplementary material for: Fungal outbreak in the Catacombs of SS. Marcellino and Pietro Rome (Italy): From diagnosis to an emergency treatment
Source: Front Microbiol. 2022 Nov 10;13:982933. doi: 10.3389/fmicb.2022.982933 (PMC9684309; doi:10.3389/fmicb.2022.982933)
Supplement: Supplementary file 2 [file Table_1.pdf]

Table 1S. Fungal strains isolation after growth on DRBC plate dishes shown in Figure 1S.

Sample	Colony colour	Identification
1W	White	*n.g.
1B	n.g.	/
2Y	n.g.	/
2B	Dark Grey	*Alternaria sp*
3W	White	*Fusarium sp.*
3Y	Green	*Penicillium sp.*
	White yeast	white yeasts
4W	Green	*Penicillium sp.*
	White/greyish	*Paecilomyces*
	Black	*Aspergillus.niger*
4B	Dark green	*Phoma sp*
	White	*Acremonium sp.*
	Green	*Penicillium sp*

n.g. no growth; *No further growth after the first isolation.
